# Modelling groundwater quality of the Athabasca River Basin in the subarctic region using a modified SWAT model

**DOI:** 10.1038/s41598-021-92920-7

**Published:** 2021-06-30

**Authors:** Tesfa Worku Meshesha, Junye Wang, Nigus Demelash Melaku, Cynthia N. McClain

**Affiliations:** 1 Athabasca River Basin Research Institute (ARBRI), Athabasca University, 1 University Drive, Athabasca, AB T9S 3A3 Canada; 2 Environment and Parks, Government of Alberta, 3535 Research Road NW, Calgary, AB T2L 2K8 Canada; 3 Department of Geoscience, University of Calgary, 2500 University Drive NW, Calgary, AB T2N 1N4 Canada

**Keywords:** Biogeochemistry, Environmental sciences, Hydrology

## Abstract

Groundwater is a vital resource for human welfare. However, due to various factors, groundwater pollution is a paramount environmental concern. It is challenging to simulate groundwater quality dynamics with the Soil and Water Assessment Tool (SWAT) because it does not adequately model nutrient percolation processes in the soil. The objectives of this study were to extend the SWAT module to simulate groundwater quality for the parameters nitrate and Total Dissolved Solids (TDS). The results of the SWAT model for the Athabasca River Basin in Canada revealed a linear relationship between observed and calculated groundwater quality. This result achieved satisfactory values for coefficient of determination (*R*^2^), Nash-Sutcliffe efficiency (*NSE*), and percent bias (*PBIAS*). For nitrate, the model performance measures *R*^2^ ranged from 0.66–0.83 during calibration and *NSE* from 0.61–0.83. *R*^2^ is 0.71 during validation and NSE ranged from 0.69–0.75. Likewise, for TDS, the model performance measures *R*^2^ ranged from 0.61–0.82 during calibration and from 0.58–0.62 during validation. When coupled with soil zone and land surface processes, nitrate and TDS concentrations in groundwater can be simulated with the SWAT model. This indicated that SWAT may be helpful in evaluating adaptive management scenarios. Hence, the extended SWAT model could be a powerful tool for regional-scale modelling of nutrient loads, and to support and effective surface and groundwater management.

## Introduction

Groundwater is a vital resource for sustainable social and economic development around the world^1,2^. Stored groundwater is generally purified and filtered during infiltration through natural soils and sediments^3,4^. Therefore, the quality and quantity of groundwater storage tends to be more stable than surface water. Thus, majority of people in the world primarily depend on groundwater for their drinking water^5^. It is reported that groundwater provides drinking water for > 1.5b people and supports approximately 40% of agriculture in the form of irrigation^6^. Therefore, protecting groundwater resources, including efficient use and conservation measures, are an important strategy of water resources plan in both developing and developed countries.

However, natural processes, anthropogenic activities, and climate change significantly influence the quality and quantity of groundwater. In many world watersheds, lakes, rivers, wetlands and the associated ecosystems have experienced impacts and thus, the vitality, availability, quality and quantity of these water resources face serious threats. Elevated concentrations of chemical elements and biological constituents exist in the environment, and depending on geo-environmental backgrounds, water pollutants may exhibit spatial and temporal variations^7,8^. Anthropogenic processes, such as discharge of untreated sewage water to water bodies, fertilization, and over exploitation, have changed groundwater quantity and quality^9–11^, leading to soil–water and air pollution^12^. For example, excessive application of chemical fertilizer could result in groundwater contamination^10,13,14^. Nitrogen leaching, namely the downward transport and percolation of nitrate from the root zone to the soil layers, is one of the aggravating causes for groundwater contamination, especially in irrigated areas^15,16^. However, it is still unclear how the anthropogenic and natural factors drive the change in the fluxes and storages of water, and associated groundwater quality. Consequently, a linked understanding of surface water and groundwater quality over space and time is critical for the assessment and management of such vital resources and ecosystems services, particularly in arid and semi-arid regions^17–20^.

Groundwater assessment is complex, involving identification of surface and groundwater quality and quantity drivers and the occurrence, interaction and determination of the probability of the occurrence of such problems^21,22^. In order to simulate both saturated and unsaturated water flow from the porous media, many widely used physical models of Richards equations has been developed by many researchers^23–25^. This approach could capture the groundwater dynamics^25^ but lacks representation of land surface information like vegetation cover. In order to address this limitation, scientists have developed various hydrological models to simulate interactions among water, soil, and vegetation^26^ modified the SWAT model to calculate lateral and vertical flows through soil layers to improve representations recharge of groundwater mountain region of Germany. Vazquez-Amábile et al.^27^ applied advanced redox zonation of an alluvial aquifer using data fusion and multivariate geostatistics. They indicated that incorrect wastewater management could cause the organic matter transfer from the eutrophicated surface–groundwater into to groundwater. Luo et al.^28^ modified SWAT model to simulate the change of soil moisture into groundwater and stream flow in the Muscatatuck River watershed. Baffaut et al.^29^ incorporated a groundwater module in the SWAT model version 2000 to simulate groundwater evaporation in the Yellow River basin. Watson et al.^30^ modified the SWAT groundwater module version 2005 to improve infiltration process from sinkholes to surface waters and to evaluate aquifer recharge in karst surroundings of the USA. Mckeown et al.^31^ and Melaku et al.^32^ incorporated an algorithm to consider the effect of slope and aspect on incoming solar radiation into the SWAT model in the forested watershed in the Boreal plain. Meshesha et al.^33^ modified the evapotranspiration in the SWAT model to account for two-way groundwater-surface water movement in estimating the groundwater table in Lethbridge and Barons area. Meshesha et al.^34^ extended the hydrological model for cold climate regions in order to quantify bacterial fluxes and its effect on surface water quality, and Kim et al.^35^ evaluated water quality consequently effect on aquatic environment. Guzman et al.^36^, Nguyen et al.^37^ and Ng et al.^38^ coupled the SWAT model with the three-dimensional groundwater flow model (MODFLOW) to represent groundwater flow. The main drawback of the coupled model is that the users must establish numerously formatted variables that properly fit^39^. Although these coupled SWAT–MODFLOW models enable simulation of groundwater recharge, aquifer evapotranspiration and groundwater levels, it is a big challenge to find a way to dynamically simulate water quality when coupling SWAT source code with MODFLOW code because the two models may have different definitions, formats and arrays of water quality variables. Therefore, such a coupled approach of the two different models requires modification of core codes, such as definitions of variables and its arrays in simulating ground water quality^40^. Although a plethora of models have been developed in the past few decades, most of the modelling experiences have focused mainly on understanding on the spatio-temporal groundwater storage. Appraisal of distribution and mitigation of chemical elements in the groundwater using SWAT model is still lacking.

Economic activity and human settlement in the Athabasca river basin (ARB) are being varied, and the basin is culturally vibrant and diverse as the homes for more than 150,000 residents with 13% of Aboriginal peoples^40^. However, the development of agriculture, recreation, forestry, conventional situ oil and gas, and mining can negatively affect the health of the river basin. Developing reliable groundwater quality model as a tool could provide a very useful insight on the potential mitigation of nonpoint source pollution into groundwater in river basins, which are especially important in assessing the pervasive high nutrients loadings from fertilization and manure application. The main objectives of this study are to: (i) improve representation of groundwater quality parameters in a groundwater nutrient module to account for nitrate (NO_3_^−^) and total dissolved solids (TDS) in the SWAT model^41^; (ii) assess the effect to NO_3_^−^ and TDS on groundwater quality status in the Athabasca River Basin; and (iii) evaluate SWAT model sensitivity and uncertainty analysis to understand the possible limitations, and recommend forthcoming directions in model formulation efforts.

## Methods and materials

### The study area

**Figure 1:**
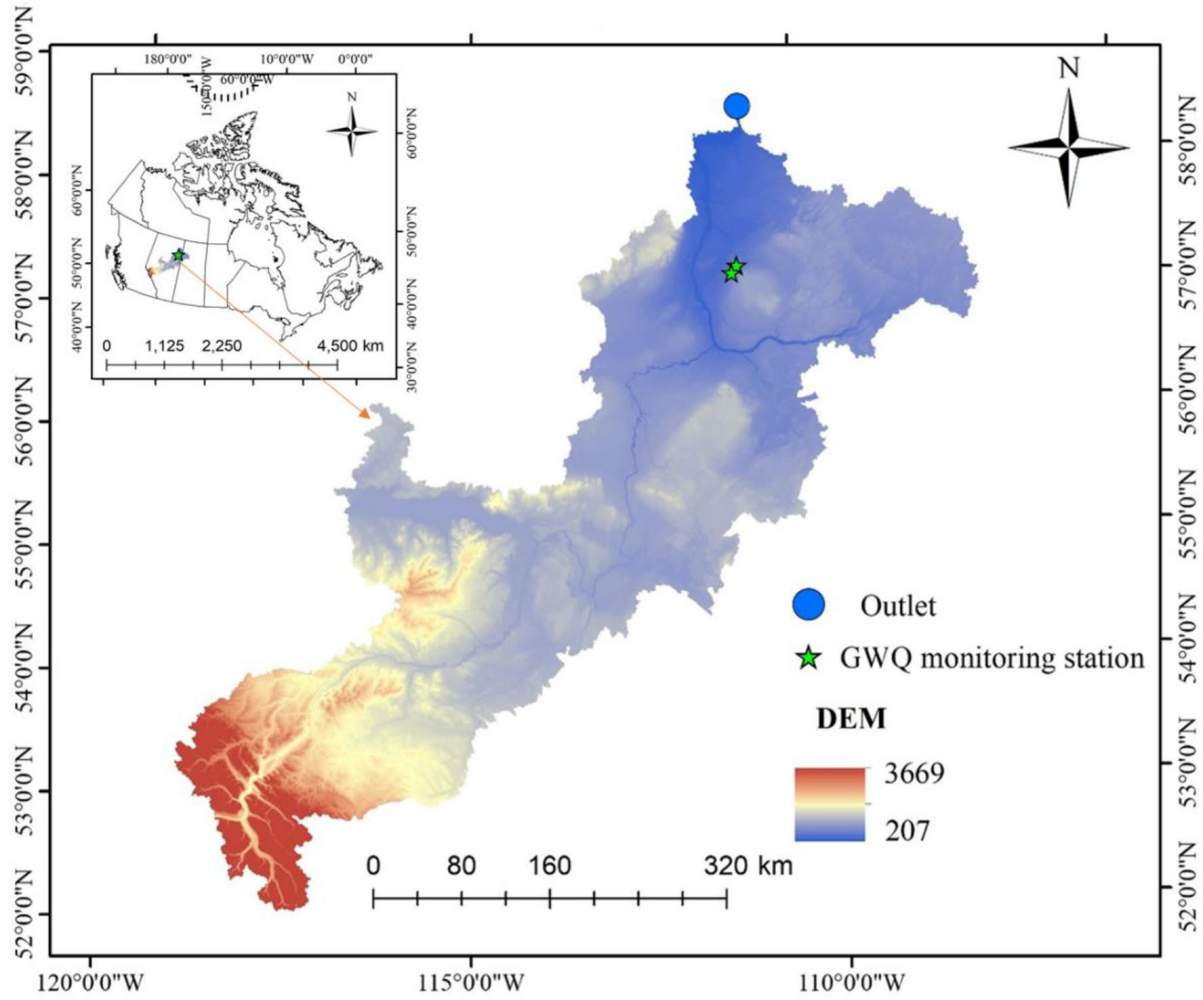
Geographical location of Athabasca River Basin (ARB), Canada. The DEM of the ARB shows the two water quality monitoring stations used for model calibration for this study. The map was generated using GIS & RS (https://www.arcgis.com/index.html).

The research area is in Athabasca River Basin (ARB), which is located in the central part of the province of Alberta in the subarctic region (Fig. 1). Athabasca River is the second largest river in Alberta, and the largest undammed river. The mean annual discharges in cubic decameters (dam^3^ = 1000 cubic meters) at the points along the river are 2,790,000 dam^3^ at Jasper 13,600,000 dam3 at Athabasca. The river basin in general have substantial economic contribution in Canada by providing reliable freshwater supply to the people as well as for various industries, such as oil sands mining and pulp mills^42^. The main land cover type of the ARB is the boreal forest, which shares 82% of the total land; agriculture shares 9.5%, which is in the central portion of the watershed (i.e., Pembina, Lesse Slave, McLeod and upper parts). Overall forest, agriculture, traditional oil and gas extraction, oil sand mining, and coal mining are the major industries of the ARB^41^. Mean annual precipitation of the area ranges from 300 mm in the lower portion of the river basin to > 1000 mm from the headwaters, while the mean temperature ranges 1.8–5.1 °C^43^.

The major aquifer types include near-surface sands, buried channels and valleys, Paskapoo aquifers, and bedrock aquifers, which characterize the basin with primary and/or secondary porosities^42^. In this area, groundwater within unconsolidated surficial deposits consist of a mixture of a preglacial and glacial sand and gravel aquifers. In the Athabasca River Basin, groundwater recharge is spatially variable, and groundwater contributes baseflow to rivers and streams^44,45^. This region has extensive wetlands, including groundwater-fed wetlands (fens), and one of the world’s most ecologically significant wetlands designated by the Ramsar Convention. The ecology within the watershed is diverse as a result of the different natural regions. As the major river system, the ARB is influenced by a variety of climate, terrain and landscape characteristics found within its basin. The seasonality of climatic conditions is a major influence on river flow conditions.

### Soil and Water Assessment Tool (SWAT) description and application

The SWAT model is a river basin scale model, which is developed in order to quantify the impact of land management practices in large, complex watersheds at daily time step^46,47^. It could be used for simulating the hydrological process, nutrient concentration, runoff generation, and sediment yield in the watershed under various landuse as well as soil scenario^46,48,49^. Additionally, it is used for simulating the effects of climate change, landuse and management practices on the quantity and quality of water^50^.

**Figure 2:**
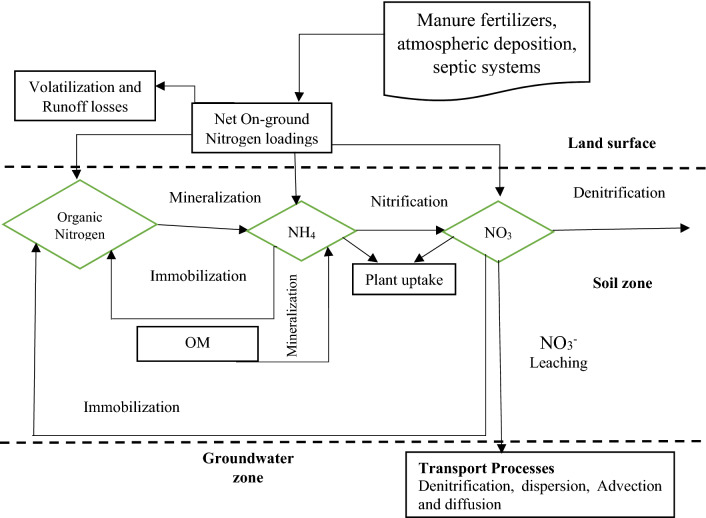
The conceptual framework demonstrating nitrate occurrence in the groundwater (adopted from Almasri^9^).

The SWAT has been used for simulating the N cycle in soils and aquifer at shallow depth^51,52^. In the water and soil, the N processes is dynamic. It could be added to the soil in the form of manure, bacteriological fixation, residue, and rainfalls. It may be moved out from the soil through by plant uptake, volatilization, soil erosion, denitrification, and leaching. In the SWAT model, there are various types of N pools, including inorganic forms of nitrogen, and organic forms. NO_3_^−^ may transport with runoff, percolation or lateral flow and recharge into the aquifer to the shallow depth from the soil profile. In addition, NO_3_^−^ can move with groundwater flow to river channels or be carried out of the shallow aquifer into the soil zone during water deficiencies. The amount of nitrate carried by water is calculated by multiplying the concentration of nitrate in the mobile water fraction by the volume of water moving in each route. The amount of NO_3_^−^ discharge depends on NO_3_^−^ concentration in the soil-water domain. Nitrate uptake by plants has an inverse exponential relationship with depth^47^.

1 $$NO_{{3conc,z}} = 7*\exp \left( {\frac{{ - z}}{{1000}}} \right)$$

where NO_3conc,z_ refers to initial nitrate concentration (mg/kg) at the depth of z (mm). Only the parts of NO_3_^−^ is mobile and therefore it available for the discharge through the tiles. To calculate the concentration of nitrate in the mobile water fraction, the following equation has been adopted from^53,54^.

2 $$Conc_{{NO_{{3,mobile}} }} = \frac{{NO_{{31y}} (1 - \exp \left[ { - W_{{mobile}} /(1 - \theta e)SAT_{{1y}} } \right])}}{{W_{{mobile}} }}$$

where ConcN_O3,mobile_ refers to NO_3_^−^ concentration in the movable water at a given layer (kg N/ha), W_mobile_ represent the amount of water lost by runoff, percolation or side flow in the layer (mm H_2_O), $\theta e$ represent fraction of porosity from which anions are excluded, SAT_1y_ is saturated water content in the soil layer (mm H_2_O). To obtain the transport of nitrate with runoff, percolation, and lateral flow, the following generic equation has been adopted from^55^.

3 $$NO_{3} = \beta _{{NO_{3} }} conc_{{NO_{{3,mobile}} }} Q_{x}$$

where NO_3_^−^ represents the NO_3_^−^ removed by the physical transport (i.e., lateral flow, runoff, percolation) (kg ha^−1^); β_NO3_ is the concentration of NO_3_^−^ in the mobile water for the top 10 mm of soil/kg ha^−1^ considering both surface and subsurface lateral flow in the top layer; and Q_x_ is the water physical transport (i.e. Q_surf_, Q_lat_, Q_perc_). The NO_3_^−^, that percolates to the shallow aquifer from the soil profile may remain in the aquifer, or it may move with groundwater flow into the main river channel or into the deep aquifer. Organic transport of N with sediment is obtained as a concentration function proposed by^56^ and later applied by^57^ to the separated runoff events. Estimating the daily organic N runoff loss is on the basis of concentration of organic N in the top soil layer, the sediment yield and the enrichment ratio of the organic nitrogen in sediment to organic N in soil layer^51^ see Fig. 2). In the SWAT model, water quality procedures integrate essential interactions and relationships used in the QUAL2E model^58^, which includes the major interactive factors such as the nutrient cycles, benthic oxygen demand, and algae production.

TDS is all the dissolved chemicals in the water (mg/L). It can be obtained by adding all the concentrations from chemical analysis or can be measured as weight of the residue after a volume of water has been evaporated to dryness. Typically, the concentration of TDS increases with the sample depth that groundwater has traveled from the recharge area to the sample sites. High nutrient concentration in the groundwater shows contamination form different sources of pollution^59^.

### Groundwater module in the SWAT model

Existing SWAT model divides underground saturated aquifer into shallow and deep aquifer. The balance of water in the shallow aquifer can be described^47,60^:

4 $$\mathop S\nolimits_{{sh}}^{i} = \mathop S\nolimits_{{sh}}^{{i - 1}} + \mathop W\nolimits_{{rg,sh}}^{i} - \mathop Q\nolimits_{{gw,sh}}^{i} - \mathop W\nolimits_{{rp}}^{i} - \mathop W\nolimits_{{pump,sh}}^{i}$$

where, $\mathop S\nolimits_{{sh}}^{i}$_and_ $\mathop S\nolimits_{{sh}}^{{i - 1}}$ is the volume of water stored in the shallow aquifer on the day i and i − 1, respectively, $\mathop Q\nolimits_{{gw,sh}}^{i}$ is refers to the groundwater from the shallow aquifer on the day *i*, $\mathop W\nolimits_{{rg,sh}}^{i}$ shows the volume of recharge which is entering the shallow aquifer on the day *i*, $\mathop W\nolimits_{{rp}}^{i}$ is indicates the amount of water entering to the soil for the evaporation as well as transpiration on the day *i*, $\mathop W\nolimits_{{pump,sh}}^{i}$ is refers to the amount of water removed from the shallow aquifer in the form of pumping on the day *i*. Assuming that the variation in groundwater is linearly correlated to the water table, the flow of groundwater can be represented by the following equations:

5 $$\mathop Q\nolimits_{{gw,sh}}^{i} = \alpha _{{gw,sh}} \mathop s\nolimits_{{sh}}^{i}$$

where, $\alpha _{{gw,sh}}$ refers to the groundwater recession constant of the shallow aquifer. Groundwater from the shallow aquifer contributed to the river on day *i* is obtained as:

6a $$\mathop Q\nolimits_{gw}^{i} = \mathop Q\nolimits_{{gw}}^{{i - 1}} \cdot \;{{e^{{- \alpha _{{gw}} \cdot \Delta t }} }} + W_{{rg,sh}} \cdot (1 - e^{{ - \alpha _{{gw}}  \cdot \Delta t}} ) \;\;\;\;if\; {S_{sh} > S_{shthr,q}}$$

6b $$\mathop Q\nolimits_{{gw}}^{i}=0 \;\;\;if\; {S_{sh} < S_{shthr,q}}$$

where $S_{shthr,q}$ is a threshold value above which the stored groundwater flows to river channels, $\alpha _{{gw}}$ is the base flow recession constant, $W_{{rg,sh}}$ is the amount of recharge entering the shallow aquifer on day *i*, and $\Delta t$ is the time step.

The volume of recharge entering the shallow aquifer on day *i* is obtained using the following equations:

7 $$\mathop W\nolimits_{{rg,dp}}^{i} = (\beta _{{dp}} )\mathop W\nolimits_{{rg}}^{i}$$

8 $$\mathop W\nolimits_{{rg,sh}}^{i} = (1-\beta _{{dp}} )\mathop W\nolimits_{{rg}}^{i}$$

9 $$\mathop W\nolimits_{{rg}}^{i} = (1- e{^{-{\frac {1}{\delta_{gw}}}}}) \mathop W\nolimits_{{seep}}^{i} + (1- e{^{-{\frac {1}{\delta_{gw}}}}}) \mathop W\nolimits_{{rg}}^{i-1} $$

where $\mathop W\nolimits_{{seep}}^{i}$ is the total amount of water exiting the bottom of the soil profile on day *i*, and $\delta_{gw}$ is the drainage time of the overlying geologic formations. $\mathop W\nolimits_{{rg}}^{i}$ and $\mathop W\nolimits_{{rg}}^{i-1}$ are the amount of recharge entering the aquifers on day *i* and *i-1*, respectively. $\delta_{gw}$ is estimated against observed data in water table level through simulating aquifer recharge.

Likewise, from the deep aquifer groundwater, which is contributed to the stream on the day, *i* is obtained by:

10 $$\mathop Q\nolimits_{{gw,dp}}^{i} = \mathop Q\nolimits_{{gw,dp}}^{{i - 1}} e^{{ - \alpha _{{gw,dp}} }} + \mathop W\nolimits_{{rg,dp}}^{i} (1 - e^{{ - \alpha _{{gw,dp}} }} )$$

where $\mathop Q\nolimits_{{gw,dp}}^{i}$ and $\mathop Q\nolimits_{{gw,dp}}^{i-1}$ refer to flow of groundwater from the deep aquifer into the stream on day *i* and *i-1*, respectively, and $\alpha_{gw,dp}$ is the groundwater recession constant of the deep aquifer.

Due to complexity of groundwater, we separate the shallow aquifer into a lower aquifer and an upper aquifer in the groundwater module of SWAT model to improve the model accuracy, similar to Shao et al.^58^. Thus, the groundwater flow in Eq. (6) can be replaced using upper and lower aquifers as follows

11 $$\mathop Q\nolimits_{{gw,u}}^{i} = \mathop Q\nolimits_{{gw,u }}^{{i - 1}} {{e^{ - u} }}+ \mathop W\nolimits_{{rg,u}}^{i} (1 - e^{{ - \alpha _{{gw,u}} }} )$$

12 $$\mathop Q\nolimits_{{gw,l}}^{i} = \mathop Q\nolimits_{{gw,l }}^{{i - 1}} {{e^{ - \alpha{_{gw,l}}} }}+ \mathop W\nolimits_{{rg,l}}^{i} (1 - e^{{ - \alpha _{{gw,l}} }} )$$

where, $\mathop Q\nolimits_{{gw,l}}^{i}$ and $\mathop Q\nolimits_{{gw,u}}^{i}$ refers to flow of groundwater from lower and upper aquifer into the stream on the day *i* respectively; where as $- \alpha _{{gw,u}}$ and $- \alpha _{{gw,l}}$ shows groundwater recession constant of the lower and upper aquifer respectively; $\mathop W\nolimits_{{rg,l}}^{i}$ and $\mathop W\nolimits_{{rg,u}}^{i}$ shows the volume of recharge entering into the lower and upper aquifer in the day *i* respectively.

### SWAT model setup

Delineation processes were done in ArcGIS. Based on soil types and landuse classes, the hydrological response unit (HRU) was defined, based on similar soil, similar landuse, and slope types. About 1370 HRUs were identified for the basins. Weather data, such as, temperature, rainfall, wind speed, humidity, and radiation observation were obtained from Alberta Environment and Parks. Groundwater well and water quality data (nitrogen species and TDS) at 1,300 different monitoring stations was obtained from Alberta Environment and Parks as shown in Fig. 1. Data availability is one of the most prominent factors which affect the model accuracy. Therefore, there were two monitoring stations with > 15 samples for the selected parameters between 2004 and 2016 namely IOR-KRL-03 (ss) and IOR-KRL-04 (ss) monitoring stations. Both groundwater monitoring wells are completed in surficial sand aquifers at 3 m and 11 m respectively.

### Model performance metrics and uncertainty analysis

Measured groundwater quality data for NO_3_^−^ and TDS were used for model calibration and validation. To evaluate model performance, coefficient of determination (*R*^2^), Nash–Sutcliffe efficiency (*NSE*), and percent bias (*PBIAS*) were used^61^. The descriptions of *NSE*, *R*^2^, and *PBIAS* can be found in^62^ as follows:

13 $$R^{2} = \left( {\frac{{\sum\limits_{{i = 1}}^{n} {(O_{i} - O_{{avr}} )(P_{i} - P_{{avr}} )} }}{{\sqrt {\sum\limits_{{i = 1}}^{n} {(O_{i} - O_{{avr}} )^{2} } } \sqrt {\sum\limits_{{i = 1}}^{n} {(P_{i} - P_{{avr}} )^{2} } } }}} \right)^{2}$$

14 $$NSE = 1 - \left[ {\frac{{\sum\limits_{{i = 1}}^{n} {(O_{i} - P_{i} )^{2} } }}{{\left[ {\sum\limits_{{i = 1}}^{n} {(O_{i} - O_{{avr}} )^{2} } } \right]}}} \right]$$

15 $$PBIAS = \left[ {\frac{{\sum\limits_{{i = 1}}^{n} {Obs_{i} - Sim_{i} )*100} }}{{\sum\limits_{{i = 1}}^{n} {Obs_{i} } }}} \right]$$

where *O*_*i*_ refers to the *i*th observed value; *O*_*avr*_ is the average observed value; *P*_*i*_ is the *i*th simulated value; *P*_*avr*_ is average simulated value; *n* is the number of time step (days in our case).

To examine the model performance, NO_3_^−^ and TDS of groundwater quality have been compared with the observed data during calibration (2009-2012) and validation (2013-2015). In order to perform parameter sensitivity and uncertainty analysis, we used Sequential Uncertainty Fitting2 (SUFI-2) algorithm in the SWAT-CUP^63^. The program generated various parameter sets for these selected parameters from a specified range of values using the Latin-Hypercube sampling technique (Table 1). To identify the total predictive uncertainty band of the simulated results, the SWATCUP was run several times, each one identifying a narrow parameter range for each parameter listed in Table 1, up to the point where reasonable goodness-of-fit statistics values were attained.

**Table 1:** The ranges of parameters included prior and after model calibration.

Parameters	input	Description	Unit	Range	Fitted value
Denitrification	CDN.bs	Denitrification exponential rate coefficient	NA	0 to 3	2.5
Ground W. Nitrn	r__GWNO3.gw	Concentration of NO_3_ in groundwater		− 1 to 1	0.5
Nitrate Percoln. co	NPERCO.bsn	Nitrate percolation coefficient		0 to 1	0.223
Transport of nitrogen growth with sediment	ERORGN.hru	Organic nitrogen enrichment ratio	NA	0 to 5	2.75
Shallow aquifer nitrate	HLIFE_NGW.gw	Half-life of nitrate-nitrogen in the shallow aquifer	Day^−1^	0 to 200	116
Mineralization	CMN.bsn	Rate factor for humus mineralization of active organic nitrogen	NA	0.0001 to 0.0003	0.000131
Nitrogen percolation	NPERCO.bsn	Percolation of nitrogen coefficient	NA	0 to 1	0.5
Nitrogen settling rate	NSETLR1.1wq	Settling nitrogen rate	m/year	1 to 150	30
N_2_ uptake	N_UPDIS.bsn	Distribution of nitrogen uptake parameter	NA	1 to 31	28
Base flow	RCHRG_DP.gw	Groundwater recharge to deep aquifer	fr	0 to 1	0.09
Base flow	REVAPMN.gw	Water depth in the shallow aquifer	mm	0 to 500	196
Base flow	GW_DELAY.gw	Groundwater delay	d	0 to 500	41
Base flow	GWQMN.gw	Threshold depth of water in the shallow aquifer required fir return flow to occur	mm	0 to 1000	618
Base flow	GW_REVAP.gw	Groundwater revap coefficient	NA	0.02 to 2	0.06
Lateral flow/infiltration	SOL_K.sol	hydraulic conductivity (Saturated)	Mmh^−1^	− 25 to + 25	16
Lateral flow/infiltration	SOL_AWC.sol	water capacity of the soil layer (Availability)	ft	− 25 to + 25	10
Runoff	CN2.mgt1	curve number for moisture condition II	NA	− 15 to + 15	10
Runoff	CH_N1.sub	Manning’s rate for tributary channel	NA	0.025 to 0.30	0.096
Base flow	ALPHA_BF.gw	Base flow alpha factor	d	0 to 1	073

## Results

### Sensitivity and uncertainty analysis

Sensitivity test analysis is to identify the most sensitive parameters that govern NO_3_^−^ and TDS in groundwater (Table 1). The range of selected sensitive parameters are presented in Table 1. Some parameters are basin scale and others are basin-based parameters. The analysis of model sensitivity can be processed to find out the relative response of the SWAT model to the changes in relative value of specific model parameters. Hence, some parameters are sensitive to control the whole system processes as the most significant parameters. Here, the most sensitive parameters governing the groundwater parameters were assessed on the basis of the values obtained during primary model calibration. The CDN.bs, r__GWNO_3_.gw, HLIFE_NGW.gw, and NPERCO.bsn were ranked as the most sensitive parameters among the NO_3_^−^ parameters. While NSETLR1.1wq and N_UPDIS.bsn were the sensitive parameters from the N parameters, that influenced the concentration. On the other hand, the parameters listed in Table 1 were the most sensitive for TDS, groundwater conditions, and surface runoff. The results of the sensitive analysis confirmed that measured input parameters have a substantial influence on the model prediction.

### Model Calibration and Validation performance

Model calibration and validation are done after the sensitivity analysis. To compare the model performance, the simulated outputs were compared with observed value. Therefore, the daily NO_3_^−^ and TDS from the groundwater monitoring stations IOR-KRL-03 (ss) and IOR-KRL-04 (ss) from ARB were employed for the model calibration and validation to evaluate the model performance using SWATCUP which was recommended by Arnold et al.^41^. Based on the criteria of model performances rating, the value of *PBIAS*, *NSE* and *R*^2^ during model calibration and validation were employed for evaluating the model performance. To calibrate the modified SWAT module, Latin-Hypercube One-factor At a Time (LH-OAT) was employed.

**Figure 3:**
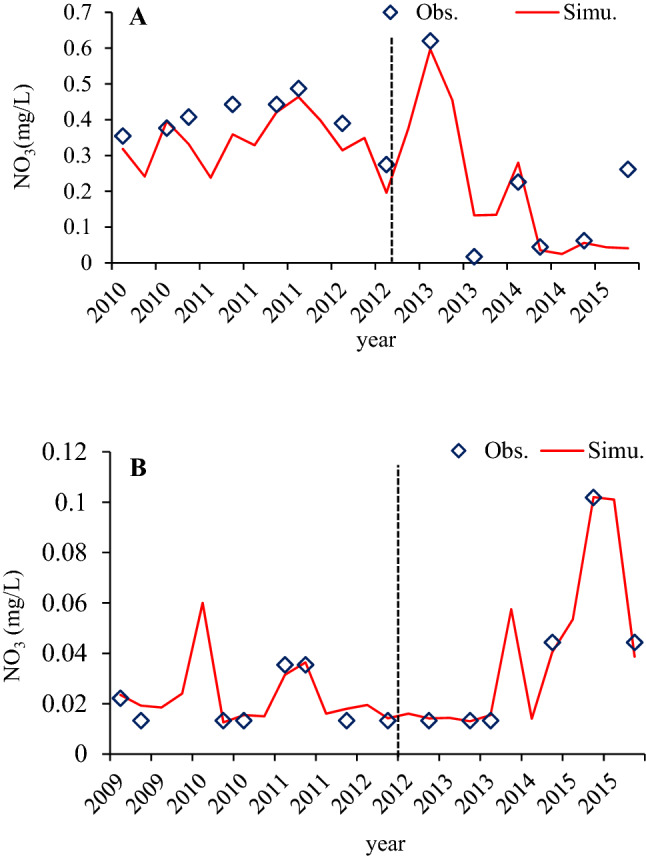
Comparison of daily observed and simulated groundwater quality parameter (NO_3_/mg/L) at IOR-KRL-03 (SS) (**A**) and IOR-KRL-04 (SS) (**B**) monitoring stations.

Table 2 summarizes the performance statistics of the model for the daily nutrient concentration simulations for two groundwater monitoring stations. Table 2 shows satisfactory to very good for both stations with an averaged *R*^2^ of 0.74 for nitrate during calibration and 0.71 during validation. This confirmed that the model was able to capture the concentration of nutrients after model modification (Table 2 and Fig. 3). Therefore, the overall model performance of the new SWAT module for the daily nutrient concentration simulations was in acceptable range of model calibration and validation in the ARB. In contrast, a lower model performance for TDS simulation was observed at both monitoring stations, for which the value of NSE is found to be 0.58 during validation at IOR-KRL-03 (ss) and 0.48 during calibration at IOR-KRL-04 (ss).

**Table 2:** Model performance assessment for the daily observed data.

Monitoring stations ID	Performance measure	NO_3_^−^		TDS	
		Calibration	Validation	Calibration	Validation
IOR-KRL-03 (ss)	NSE	0.61	0.69	0.72	0.58
	PBIAS	7.32	6.31	9.34	−6.72
	R^2^	0.66	0.71	0.82	0.69
IOR-KRL-04 (ss)	NSE	0.83	0.75	0.48	0.62
	PBIAS	11.32	6.19	10.04	6.06
	R^2^	0.83	0.71	0.61	0.67

### Nitrate (NO_3_^−^)

Nitrate calibration processes happened in a nitrogen percolation coefficient of 0.5, with a denitrification threshold of water content and exponential rate of denitrification coefficient of 2.5 (Table 1). Nitrate percolation coefficient (0.223) and half-life parameter of nitrate in the shallow aquifer (116) were calibrated for each sub-basin for the values in the range of 0–1 and 0–200 day^−1^ respectively, resulting in 0.223 and with the mean value of 116 day^−1^ respectively for the whole selected ARB. The annual mean groundwater nitrate concentration is 0.31 mg/L at station IOR-KRL-03(ss) and 0.03 mg/L at station IOR-KRL-04(ss) (Fig. 3). Mineralization was adjusted by minimizing the default values of the rate factor for CMN to 0.000131. To control the depth distribution of nitrogen uptake, and to slow down simulated kinetics, the N-UPDIS was increased from the default value of 20 to 28 and therefore a large mass of NO_3_^−^ was removed from the upper layers as per the report by^64,65^. The range of nitrogen settling in reservoirs was kept constant during the year as per^66^, the range was set greater than the default values from the Sava River Basin, to efficiently simulate the substantial retention in major wetlands because wetland specific retention is not applied in the extended SWAT model.

The nitrification rate decreased from the upper to lower Athabasca River Basin, which mirrors the rainfall distribution, with lower rainfall leading to lower soil saturation and lower nitrification rates. The maximum values of nitrate in groundwater of the ARB were 0.62 mg/L at IOR-KRL-03(ss) and 0.1 mg/L at IOR-KRL-04(ss) stations respectively, whereas the minimum values were 0.02 mg/L at the IOR-KRL-03(ss) station and 0.01 mg/L at the IOR-KRL-04(ss) station. The nitrification calibration resulted to satisfactory predictions of NO_3_^−^ daily concentration in the two observation stations in the calibration and validation dataset. Ae per the comparison of NO_3_^−^ between simulated and observed, better model performances were obtained in the model evaluation dataset. The simulated daily NO_3_^−^ concentration agreed with the observed data and were acceptable range (Fig. 3). The percentage BIAS obtained from the observed and calculated daily loads also was ranked as acceptable to very good for all the selected stations (Fig. 3 and Table 2).

### Total dissolved solids (TDS)

**Figure 4:**
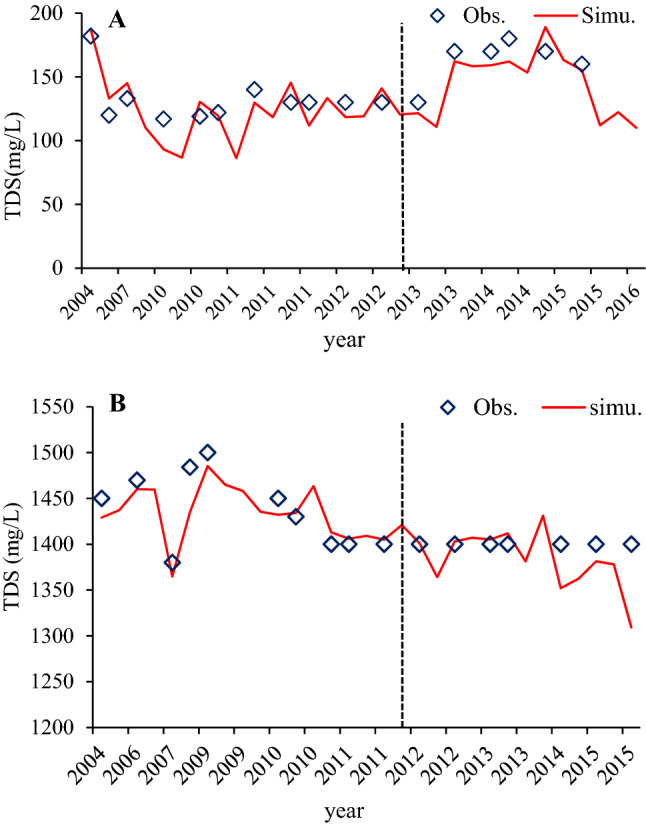
Comparison of daily observed and simulated groundwater quality parameter (TDS/mg/L) at IOR-KRL-03 (SS) (**A**) and IOR-KRL-04 (SS) (**B**) monitoring stations.

The TDS module for groundwater quality used the TDS concentrations from the selected groundwater monitoring stations in the ARB. The calibrated TDS parameters with specific ranges were presented in Table 1. Daily TDS concentrations were calibrated by adjusting the parameters related to groundwater. The model performance evaluation criteria reported by^62^ were used for the daily nutrient simulation as a guideline in evaluating the model performance for the daily TDS concentrations. Figure 4 shows that for the whole period of simulation, R^2^, NSE and PBIAS values were found to be good to very good during calibration while they were satisfactory to good during the validation (Table 2). Generally, the overall model performance for the daily TDS concentration simulations in groundwater of the ARB shows that the model could capture the observed concentrations. When comparing the calculated and observed concentrations (Fig. 4), the simulated TDS concentrations were also acceptable as both show similar trends. Yet, local inconsistencies were noticed in the river basin. The highest percentage of overestimation and underestimation of TDS in the calibration dataset occurs for high concentration observations and probably reflects the SWAT model’s representation of high level concentrations^67^, which may cause errors in the process of calculating TDS in the SWAT model^68^. The other possible sources of overestimation and underestimation of the model simulations originate from uncertainties in the input data and observed data. For example, the frequency of data collection varied from once or twice per month to once every few years. In contrast, the simulations used a daily time step. The low frequency of sampling might miss the peak or valley of nutrient concentrations.

Total Dissolved Solids is an indicator of the availability of total dissolved salt and other constituents that affect groundwater quality. High TDS concentrations in groundwater can occur naturally, and may also indicate a downward movement of leachate into groundwater^65,69–71^. The average TDS concentrations in groundwater in the study area are 143 mg/L and 1421 mg/L at IOR-KRL-03(ss) and IOR-KRL-03(ss) stations, respectively. The TDS of groundwater samples ranges from 120-182 mg/L at the IOR-KRL-03(ss) station, while the TDS ranges from 1380-1500 mg/L at the IOR-KRL-04(ss) station (Fig. 4). High concentrations of TDS in groundwater reduce the palpability of water for drinking and may cause gastrointestinal pain and emetic effects in humans^67^. TDS is an important indicator for evaluating the quality of groundwater. High levels of TDS typically occur for hard water and might require groundwater treatment to decrease concentrations below 500 mg/L^67^.

**Figure 5:**
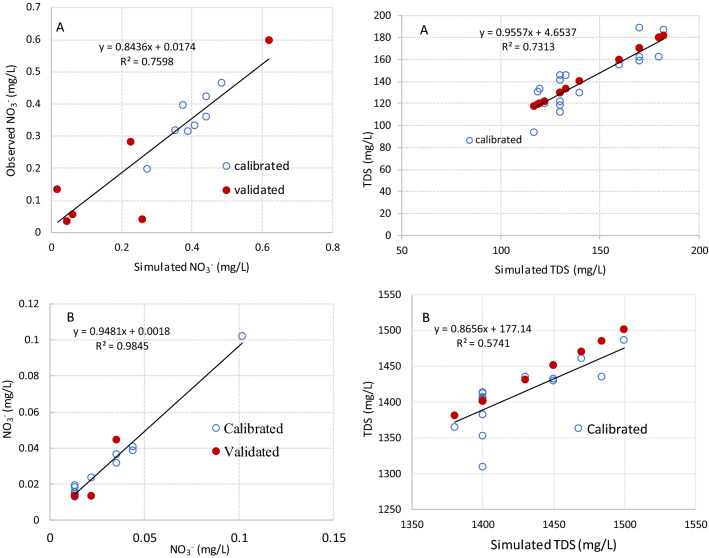
Scatter plot comparison between of daily simulated and observed groundwater quality parameters at IOR-KRL-03 (SS) (**A**) and IOR-KRL-04 (SS) (**B**) monitoring stations.

The performance of model results were investigated by using the analysis of scatter plot between observed and simulated estimates at each groundwater monitoring stations (Fig. 5). The slope at the selected stations were considerably far from zero. This showed that the model prediction accuracy was enhanced by extended groundwater model. Thus, the results of the 1:1 fitting line confirmed that the extended groundwater SWAT module was effective prediction of nutrient concentration in the groundwater. The scatters are closer to the 1:1 line for the entire study time at two monitoring stations, albeit at some points relatively far from the fitting line probably due to limited availability of observed data. In general, it is worth to conclude the extended SWAT groundwater module shows better efficiency for simulating nutrient concentrations in the groundwater.

### Groundwater quality analysis and nutrient concentrations

Analysis of observed against simulated nitrate and TDS concentrations in ARB groundwater helps to identify sources of errors across two groundwater monitoring stations within the river basin. Observed nitrate concentrations vary significantly across the area (Fig. 3). Concentrations of NO_3_^−^ in groundwater have been highly affected by emissions of both point and non-point sources in different watersheds across the world^71^. To assess pollution sources and quantify the loads of NO_3_^−^ entering the whole river basin, NO_3_^−^ load to groundwater has been simulated at various hydrogeological response units. The simulation periods were chosen based on the availability of point-source information. The long-term daily average concentrations of nitrate in groundwater attained maximum values of 0.59 mg/L at the IOR-KRL-03(ss) station during 2013. The long-term daily average concentrations of nitrate in groundwater reached a minimum of 0.025 mg/L in the year 2014. On the other hand, at station at IORKRL-04(ss) the long-term daily average NO_3_^−^ concentrations recorded a maximum value of 0.1 mg/L during the year 2015, and a minimum value of 0.013 mg/L in most observation years. Generally, the concentrations of nitrate were well captured in all the groundwater monitoring stations, although some overestimations were observed at some points at IOR-KRL-04(ss) monitoring station (Fig. 3) and some underestimations were found at IOR-KRL-03(ss) and IOR-KRL-04(ss) monitoring stations. The probable reason for model overestimation is the data uncertainty such rainfall and snow distribution, in which the rainfall and snow distribution caused in nutrient concentrations. The Underestimation was probably subjected to the uncertainty of the input data and measured data. Furthermore, the observed NO_3_^−^ data is not sufficient for continuous daily NO_3_^−^ concentration for the entirely considered period for this study. Therefore, observed data could be sources for the errors. The simulation of NO_3_^−^ removal by soil denitrification could be improved by varying the river basin parameters. However, the existing SWAT model could not represent perfectly the seasonal difference in nitrate concentrations. After carefully extending the model, the findings of this study highlight the need to improve spatial representation of nitrate concentrations in groundwater and in the parameters that influence nitrate concentrations at the watershed scale.

## Concluding remarks

Groundwater is a precious natural resource supporting the existence of life on earth. However, various factors, such as soil properties, crop growth, industrial wastes, and agricultural management, can influence its quality. Development of industries and agriculture increase water use, which puts pressure on groundwater quality and may, in turn, influence the ecosystem of the Athabasca River Basin. Particularly, the use of fertilizers, manure, and industrial wastes may contribute to the pollution of groundwater and connected surface water environments. It is necessary to perform optimal management of groundwater resources in the basin.

State-of-the-art groundwater quality modelling at the ARB is recognized as a vital component of groundwater management, where further in-depth studies may be required to offer valuable insights related to groundwater condition and nutrient processes at the various spatio-temporal scales (i.e. site and region, and daily, monthly and annually). This can better support nutrient monitoring networks for river basin management, and enhance understanding of changing nutrient concentrations in the ARB. The SWAT model results could support the development of indicators for groundwater quality parameters, and also support integrated surface water and groundwater management. However, the accuracy of SWAT predictions is limited by data availability and structure of the model. This may result in errors while modeling processes in the river basin. For example, the highest percentage of overestimation and underestimation for TDS in the calibration dataset probably reflected the poor representation of SWAT model to high concentrations, as well as uncertainties of the input data and observed data, which in turn may cause errors in estimating groundwater TDS in the SWAT model. With continued monitoring of the groundwater network at regular frequencies, high quality input data would be available for better calibration and validation of the model. Furthermore, additional data would improve the representation of processes and pathways controlling groundwater pollution and therefore allow evaluation of the effect of land and water management practices to support the implementation of the best management practices.

In this study, we extended the existing SWAT model to improve modelling of groundwater quality (i.e. NO_3_^−^ and TDS). A systematic calibration and validation of the SWAT model has been performed to compare the observed groundwater NO_3_^−^ and TDS concentrations in the ARB. The results revealed that the new groundwater quality model in the SWAT is able to capture the daily nutrient concentrations in groundwater. The simulated results agree with the observed data with satisfactory and good performance for both groundwater monitoring stations as per the model performance measures. Thus, the process-based hydrologic groundwater quality model is an effective tool in simulating the groundwater quality dynamics (NO_3_^−^ and TDS) for sustainable groundwater and surface water management in the river basin.
