# Publisher Correction: Modelling groundwater quality of the Athabasca River Basin in the subarctic region using a modified SWAT model

**DOI:** 10.1038/s41598-022-06145-3

**Published:** 2022-03-29

**Authors:** Tesfa Worku Meshesha, Junye Wang, Nigus Demelash Melaku, Cynthia N. McClain

**Affiliations:** 1 Athabasca River Basin Research Institute (ARBRI), Athabasca University, 1 University Drive, Athabasca, AB T9S 3A3 Canada; 2 Environment and Parks, Government of Alberta, 3535 Research Road NW, Calgary, AB T2L 2K8 Canada; 3 Department of Geoscience, University of Calgary, 2500 University Drive NW, Calgary, AB T2N 1N4 Canada

Correction to: *Scientific Reports* 10.1038/s41598-021-92920-7, published online 30 June 2021

The original version of this Article contained errors introduced due to miscommunication during the proofing process. These mistakes include grammatical errors as well as changes to figures, equations and one table.

Cynthia N. McClain was incorrectly affiliated with ‘Resource Stewardship Division, Alberta Environment and Parks, 3535 Research Road NW, Calgary, AB, T2L 2K8, Canada’ and ‘University of Calgary, 3535 Research Road NW University Park Calgary, Alberta, T2L 2K8, Canada’. The correct affiliation is listed below.

Environment and Parks, Government of Alberta, 3535 Research Road NW, Calgary, AB T2L 2K8, Canada.

Department of Geoscience, University of Calgary, 2500 University Drive NW, Calgary, AB T2N 1N4, Canada.

In the Abstract,

“However, due to various factors, groundwater pollution is one of the main environmental concerns. Yet, it is challenging to simulate groundwater quality dynamics due to the insufficient representation of nutrient percolation processes in the soil and Water Assessment Tool model. The objectives of this study were extending the SWAT module to predict groundwater quality. The results proved a linear relationship between observed and calculated groundwater quality with coefficient of determination (*R*^2^), Nash–Sutcliffe efficiency (*NSE*), percent bias (*PBIAS*) values in the satisfied ranges. While the values of *R*^2^, *NSE* and *PBIAS* were 0.69, 0.65, and 2.68 during nitrate calibration, they were 0.85, 0.85 and 5.44, respectively during nitrate validation. Whereas the values of *R*^2^, *NSE* and *PBIAS* were 0.59, 0.37, and − 2.21 during total dissolved solid (TDS) calibration and they were 0.81, 0.80, 7.5 during the validation. The results showed that the nitrate and TDS concentrations in groundwater might change with varying surface water quality. This indicated the requirement for designing adaptive management scenarios. Hence, the extended SWAT model could be a powerful tool for future regional to global scale modelling of nutrient loads and effective surface and groundwater management.”

now reads:

“However, due to various factors, groundwater pollution is a paramount environmental concern. It is challenging to simulate groundwater quality dynamics with the Soil and Water Assessment Tool (SWAT) because it does not adequately model nutrient percolation processes in the soil. The objectives of this study were to extend the SWAT module to simulate groundwater quality for the parameters nitrate and Total Dissolved Solids (TDS). The results of the SWAT model for the Athabasca River Basin in Canada revealed a linear relationship between observed and calculated groundwater quality. This result achieved satisfactory values for coefficient of determination (*R*^*2*^), Nash-Sutcliffe efficiency (*NSE*), and percent bias (*PBIAS*). For nitrate, the model performance measures *R*^*2*^ ranged from 0.66–0.83 during calibration and NSE from 0.61–0.83. *R*^*2*^ is 0.71 during validation and *NSE* ranged from 0.69–0.75. Likewise, for TDS, the model performance measures *R*^*2*^ ranged from 0.61–0.82 during calibration and from 0.58–0.62 during validation. When coupled with soil zone and land surface processes, nitrate and TDS concentrations in groundwater can be simulated with the SWAT model. This indicated that SWAT may be helpful in evaluating adaptive management scenarios. Hence, the extended SWAT model could be a powerful tool for regional-scale modelling of nutrient loads, and to support and effective surface and groundwater management.”

In the Introduction,

“The main objectives of this study are to: (i) improve water carrying capacity of groundwater quality parameters in a groundwater nutrient module to account for nitrate (NO_3_^−^) and total dissolved solid (TDS) in the SWAT model;”

now reads:

“The main objectives of this study are to: (i) improve representation of groundwater quality parameters in a groundwater nutrient module to account for nitrate (NO_3_^−^) and total dissolved solids (TDS) in the SWAT model^41^;”

In the Methods and materials section, under the subheading ‘The study area’,

“Groundwater could recharge to stream flow in the Athabasca River are^44,45^. In-situ oil sands operations typically occur where soil sand deposits in the southern Athabasca Oil Sands region. It is one the world’s most ecologically significant wetlands and has been designated as a Ramsar Convention wetland.”

now reads:

“In the Athabasca River Basin, groundwater recharge is spatially variable, and groundwater contributes baseflow to rivers and streams^44,45^. This region has extensive wetlands, including groundwater-fed wetlands (fens), and one of the world’s most ecologically significant wetlands designated by the Ramsar Convention.”

Under the subheading ‘Soil and Water Assessment Tool (SWAT) description and application’,

“Also NO_3_^−^ could be moved with the groundwater flow through main channel or it could be carried out of the shallow aquifer by water into the soil zone during water deficiencies. The amount of nitrate carried by the water is calculated by multiplying the concentration of nitrate in the mobile water by the volume of water moving in each route. The amount of NO_3_^−^ discharge into the tiles depends on NO_3_^−^ concentration in the soil–water levels. The first level of nitrate uptake by plants, which is the initial form of nitrogen_,_ have inverse exponential relationship with the depth^47^.”

now reads:

“In addition, NO_3_^−^can move with groundwater flow to river channels or be carried out of the shallow aquifer into the soil zone during water deficiencies. The amount of nitrate carried by water is calculated by multiplying the concentration of nitrate in the mobile water fraction by the volume of water moving in each route. The amount of NO_3_^−^ discharge depends on NO_3_^−^ concentration in the soil-water domain. Nitrate uptake by plants has an inverse exponential relationship with depth^47^.”

“NO_3_^−^, which is percolated to the shallow aquifer from the soil profile, may store in the aquifer or move with groundwater flow into the main channel or into the shallow aquifer and recharge the deep aquifer.”

now reads:

“The NO_3_^−^ that percolates to the shallow aquifer from the soil profile may remain in the aquifer, or it may move with groundwater flow into the main river channel or into the deep aquifer.”

Under the subheading ‘Groundwater module in the SWAT model’,

“Assuming the there is no pumping and no re-evaporation, is employed to calculate the flow of groundwater using the following equation:

5 $$Q_{gw,sh}^{i} = \alpha_{gw,sh} s_{sh}^{i}$$

where, $\alpha_{gw,sh}$ refers to the groundwater recession constant of the shallow aquifer. From groundwater from the shallow aquifer contributed for stream on the day *i* is obtained as:

6 $$Q_{gw,sh}^{i} = Q_{{gw,sh^{{e^{{\alpha_{gw,sh} }} }} }}^{i - 1} + W_{rg,sh}^{i} \left( {1 - e^{{ - \alpha_{gw,sh} }} } \right)$$

The volume of recharge entering to the shallow aquifer on the day *i* is obtained using the following equation:

7 $$W_{rg,sh} = \left( {1 - \beta_{dp} } \right)W_{rg}^{i}$$

8 $$W_{rg}^{i} - ( {1 - e^{{ - \frac{1}{{\delta_{gw,sh} }}}} })W_{seep} + e^{{ - \frac{1}{{\alpha_{gw,sh} }}W_{rg}^{i - 1} }}$$

9 $$W_{rg,deep} = \beta_{dp} W_{rg}^{i}$$

where, $W_{rg}^{i}$ and $W_{rg}^{i - 1}$ refers the volume of recharge which is entering into the aquifers (onto both shallow and deep) on the day *i* and *i* − 1 respectively, $\delta_{gw,sh}$ refers to the coefficient that shows the delay time of the recharge for the shallow aquifer, $W_{rg,deep}$ shows the volume of water exiting the bottom of soil profile on the day *i*, and $\beta_{dp}$ shows the coefficient which reflects the volume of water percolated into deep aquifer. Likewise, from the deep aquifer groundwater, which is contributed to the stream on the day, *i* is obtained by:

10 $$Q_{gw,deep}^{i} = Q_{gw,deep}^{i - 1} e^{{ - \alpha_{gw,deep} }} + W_{rg,deep}^{i} \left( {1 - e^{{ - \alpha_{gw,deep} }} } \right)$$

In order to represent better simulation results of groundwater modelling, we adopted Eq. (6) as follows:

11 $$Q_{gw,u}^{i} = Q_{{gw,u^{{e^{{ - \alpha_{gw,u} }} }} }}^{i - 1} + W_{rg,u}^{i} \left( {1 - e^{{ - \alpha_{gw,u} }} } \right)$$

12 $$Q_{gw,l}^{i} = Q_{{gw,l^{{e^{{ - \alpha_{gw,l} }} }} }}^{i - 1} + W_{rg,l}^{i} \left( {1 - e^{{ - \alpha_{gw,l} }} } \right)$$

where, $Q_{gw,l}^{i}$ and $Q_{gw,u}^{i}$ refers to flow of groundwater from lower and upper aquifer into the stream on the day *i* respectively; where as $- \alpha_{gw,u}$ and $- \alpha_{gw,l}$ shows groundwater recession constant of the lower and upper aquifer respectively; $W_{rg,l}^{i}$ and $W_{rg,u}^{i}$ shows the volume of recharge entering into the lower and upper aquifer in the day *i* respectively.”

now reads:

“Assuming that the variation in groundwater is linearly correlated to the water table, the flow of groundwater can be represented by the following equations:

5 $$Q_{gw,sh}^{i} = \alpha_{gw,sh} \mathop s\nolimits_{{sh}}^{i}$$

where, $\alpha_{gw,sh}$ refers to the groundwater recession constant of the shallow aquifer. Groundwater from the shallow aquifer contributed to the river on day *i* is obtained as:

6a $$Q_{gw}^{i} = Q_{gw}^{i - 1} \cdot e^{{ - \alpha_{gw} \cdot \Delta t}} + W_{rg,sh} \cdot \left( {1 - e^{{ - \alpha_{gw} \cdot \Delta t}} } \right)\quad if\quad S_{sh} > S_{shthr,q}$$

6b $$Q_{gw}^{i} = 0\quad if\quad S_{sh} < S_{shthr,q}$$

where *S*_*shthr,q*_ is a threshold value above which the stored groundwater flows to river channels, *α*_*gw*_ is the base flow recession constant, *W*_*rg,sh*_ is the amount of recharge entering the shallow aquifer on day *i*, and Δt is the time step.

The volume of recharge entering the shallow aquifer on day *i* is obtained using the following equations:

7 $$W_{rg,dp}^{i} = \beta_{dp} W_{rg}^{i}$$

8 $$W_{rg,sh}^{i} = \left( {1 - \beta_{dp} } \right)W_{rg}^{i}$$

9 $$W_{rg}^{i} = ( {1 - e^{{ - \frac{1}{{\delta_{gw} }}}} })W_{seep}^{i} + ( {1 - e^{{ - \frac{1}{\delta }_{gw} }} })W_{rg}^{i - 1}$$

where $W_{seep}^{i}$ is the total amount of water exiting the bottom of the soil profile on day *i*, and $\delta_{gw}$ is the drainage time of the overlying geologic formations. $W_{rg}^{i}$ and $W_{rg}^{i - 1}$ are the amount of recharge entering the aquifers on day *i* and *i* − *1*, respectively. $\delta_{gw}$ is estimated against observed data in water table level through simulating aquifer recharge.

Likewise, deep aquifer groundwater, which is contributed to the stream on day, *i* is obtained by:

10 $$Q_{gw,dp}^{i} = Q_{gw,dp}^{i - 1} e^{{ - \alpha_{gw,dp} }} + W_{rg,dp}^{i} \left( {1 - e^{{ - \alpha_{gw,dp} }} } \right)$$

where $Q_{gw,dp}^{i}$ and $Q_{gw,dp}^{i - 1}$ refer to flow of groundwater from the deep aquifer into the stream on day *i* and *i-1,* respectively, and $\alpha_{gw,dp}$ is the groundwater recession constant of the deep aquifer.

Due to complexity of groundwater, we separate the shallow aquifer into a lower aquifer and an upper aquifer in the groundwater module of SWAT model to improve the model accuracy, similar to Shao et al.^58^. Thus, the groundwater flow in Eq. (6) can be replaced using upper and lower aquifers as follows

11 $$Q_{gw,u}^{i} = Q_{gw,u}^{i - 1} e^{ - u} + W_{rg,u}^{i} \left( {1 - e^{{ - \alpha_{gw,u} }} } \right)$$

12 $$Q_{gw,l}^{i} = Q_{gw,l}^{i - 1} e^{{ - \alpha_{gw,l} }} + W_{rg,l}^{i} \left( {1 - e^{{ - \alpha_{gw,l} }} } \right)$$

where, $Q_{gw,l}^{i}$ and $Q_{gw,u}^{i}$ refers to flow of groundwater from lower and upper aquifer into the stream on the day *i* respectively; where as $- \alpha_{gw,u}$ and $- \alpha_{gw,l}$ shows groundwater recession constant of the lower and upper aquifer respectively; $W_{rg,l}^{i}$ and $W_{rg,u}^{i}$ shows the volume of recharge entering into the lower and upper aquifer in the day *i* respectively.”

Additionally, there is a repeated error where the data for the two monitoring stations was incorrect. The monitoring stations, IOR-KRL-03 (ss) and IOR-KRL-04 (ss) replaced the stations ABG021546 and ABG022048.

As a result, in the Methods and materials section, under the subheading ‘SWAT model setup’,

“Therefore, there were three monitoring stations with > 15 samples for the selected parameters between 1986 and 2003 namely Petro Canada Main Camp GOWN #256 (ABG0215416), House River 2193E GOWN#207 & House River 2194E (NE) GOWN #257 (ABG0220481).”

now reads:

“Therefore, there were two monitoring stations with > 15 samples for the selected parameters between 2004 and 2016 namely IOR-KRL-03 (ss) and IOR-KRL-04 (ss) monitoring stations. Both groundwater monitoring wells are completed in surficial sand aquifers at 3 m and 11 m respectively.”

The subheading ‘Model performance metrix and uncertainty analysis’ has been renamed to ‘Model performance metrics and uncertainty analysis’.

Under the subheading ‘Model performance metrics and uncertainty analysis’,

“Measured daily groundwater quality data of NO_3_^−^ and TDS were used for the model calibration and validation.”

now reads:

“Measured groundwater quality data for NO_3_^−^ and TDS were used for model calibration and validation.”

“To examine the model performance, NO_3_^−^ and TDS of groundwater quality have been compared with the observed data during calibration (1986–1997) and validation (1998–2003).”

now reads:

“To examine the model performance, NO_3_^−^ and TDS of groundwater quality have been compared with the observed data during calibration (2009–2012) and validation (2013–2015).”

In the Results section, under the subheading ‘Model Calibration and Validation performance’,

“Therefore, the daily NO_3_^−^ and TDS from the groundwater monitoring stations ABG0215416 and ABG0220481 from ARB were employed for the model calibration and validation to evaluate the model performance using SWATCUP which was recommended by Arnold et al. (2012).”

now reads:

“Therefore, the daily NO_3_^−^ and TDS from the groundwater monitoring stations IOR-KRL-03 (ss) and IOR-KRL-04 (ss) from ARB were employed for the model calibration and validation to evaluate the model performance using SWATCUP which was recommended by Arnold et al. (2012)^41^.”

“Table 2 summaries the performance statistics of the model for the daily nutrient concentration simulations for two stations. Table 2 shows satisfactory to very good for both stations with an averaged *R*^2^ of 0.77 for nitrate during calibration and 0.68 during validation. This confirmed that the model was able to capture the concentration of nutrients after model modification (Table 2 and Fig. 3). Therefore, the overall model performance of the new SWAT module for the daily nutrient concentration simulations was in acceptable range of model calibration and validation in the ARB. However, a lower model performance for TDS simulation was observed at monitoring station ABG0220481, in which the value of NSE is found to be 0.37.”

now reads:

“Table 2 summarizes the performance statistics of the model for the daily nutrient concentration simulations for two groundwater monitoring stations. Table 2 shows satisfactory to very good for both stations with an averaged *R*^*2*^ of 0.74 for nitrate during calibration and 0.71 during validation. This confirmed that the model was able to capture nutrients concentration in groundwater after model modification (Table 2 and Fig. 3). Therefore, the overall model performance of the new SWAT module for the daily nutrient concentration simulations was in an acceptable range for model calibration and validation in groundwater of the ARB. In contrast, a lower model performance for TDS simulation was observed at both monitoring stations, for which the value of NSE is found to be 0.58 during validation at IOR-KRL-03 (ss) and 0.48 during calibration at IOR-KRL-04 (ss).”

In Table 2, the Monitoring stations IDs and corresponding data were incorrect. The correct and incorrect values appear below.

Incorrect:

**Table:** 

Monitoring stations ID	Performance measure	NO_3_^−^		TDS	
		Calibration	Validation	Calibration	Validation
ABG0215416	NSE	0.65	0.72	0.80	0.55
	PBIAS	2.68	3.41	− 2.21	0.43
	R^2^	0.69	0.66	0.81	0.66
ABG0220481	NSE	0.85	0.73	0.37	0.66
	PBIAS	5.44	8.02	5.36	7.5
	R^2^	0.85	0.70	0.59	0.69

Correct:

**Table:** 

Monitoring stations ID	Performance measure	NO_3_^−^		TDS	
		Calibration	Validation	Calibration	Validation
IOR-KRL-03 (ss)	NSE	0.61	0.69	0.72	0.58
	PBIAS	7.32	6.31	9.34	− 6.72
	R^2^	0.66	0.71	0.82	0.69
IOR-KRL-04 (ss)	NSE	0.83	0.75	0.48	0.62
	PBIAS	11.32	6.19	10.04	6.06
	R^2^	0.83	0.71	0.61	0.67

Under the subheading ‘Nitrate (NO_3_^−^)’,

“The annual mean nitrification was thus estimated for the selected basin observed around 0.164 mg/L at station ABG0215416 and 0.116 mg/L at station ABG0220481 stations (Fig. 3). Since NO_3_^−^ is one forms of nitrogen, the parameters were adjusted that affect N_2_. Therefore, the processes of mineralization were adjusted by minimizing the default values of the rate factor of CMN to 0.000131 and the percolation coefficient of nitrogen to 0.5. To slow down simulated kinetics, for better controlling the depth distribution of nitrogen uptake the N-UPDIS has been increased the default value from 20 to 28 and therefore large volume of NO_3_^−^ removed form the upper layers as per the report by^64,65^. The rate of nitrogen settling in reservoirs were kept steady during the year and as per^66^, the range has set greater than the default values as of in the SRB (Sava River Basin), to efficiently simulate the substantial retention of major wetlands not applied in the extended SWAT model.

The nitrification dwindled from upper to lower Athabasca River Basin, which signifies the rainfall distribution subsequently lower rainfall, leads to lower soil saturation then consequently lower nitrification. The maximum values of annual nitrification in the ARB is found to be 0.46 mg/L at ABG0215416 and 0.29 mg/L at ABG022048 stations respectively, whereas the minimum values were 0.012 mg/L in the ABG0215416 station and 0.001 mg/L generally observed in the ABG0220481stations.”

now reads:

“The annual mean groundwater nitrate concentration is 0.31 mg/L at station IOR-KRL-03(ss) and 0.03 mg/L at station IOR-KRL-04(ss) (Fig. 3). Mineralization was adjusted by minimizing the default values of the rate factor for CMN to 0.000131. To control the depth distribution of nitrogen uptake, and to slow down simulated kinetics, the N-UPDIS was increased from the default value of 20 to 28 and therefore a large mass of NO_3_^−^ was removed from the upper layers as per the report by^64,65^. The range of nitrogen settling in reservoirs was kept constant during the year as per^66^, the range was set greater than the default values from the Sava River Basin, to efficiently simulate the substantial retention in major wetlands because wetland specific retention is not applied in the extended SWAT model.

The nitrification rate decreased from the upper to lower Athabasca River Basin, which mirrors the rainfall distribution, with lower rainfall leading to lower soil saturation and lower nitrification rates. The maximum values of nitrate in groundwater of the ARB were 0.62 mg/L at IOR-KRL-03(ss) and 0.1 mg/L at IOR-KRL-04(ss) stations respectively, whereas the minimum values were 0.02 mg/L at the IOR-KRL-03(ss) station and 0.01 mg/L at the IOR-KRL-04(ss) station.”

Under the subheading ‘Total dissolved solids (TDS)’,

“The development of TDS module for groundwater quality used the daily TDS concentration from the selected stations at the ARB. The calibrated TDS parameters with specific ranges were presented in Table 1. Daily dissolved solid loads were calibrated by correcting the parameters settling to the groundwater. Some of the parameters which control groundwater TDS concentration are considered to be HRU scale while the other parameters are considered to basin scale. The model performance evaluation criteria reported by^62^ were used for the daily nutrient simulation as guideline in evaluating the model performance for the daily TDS loads. Figure 4 shows that the whole periods of simulation, R^2^, NSE and PBIAS values were found to be good to very good during calibration while they were satisfactory to good during the validation (Table 2). Generally, the overall model performance for the daily TDS loads simulations in the ARB shows that the model could capture the observed loads. On the basis comparison between the calculated and observed daily concentrations (Fig. 4), the TDS loads were also acceptable as both shows similar trend. Yet, local inconsistencies were noticed through the river basin. The highest percentage of overestimation and underestimation performance for the TDS in the calibration dataset probably reflected the representation of SWAT model to high-level concentrations^67^, which in turns may cause errors in process of estimating the TDS in the SWAT model. On the other hand, the lower model performance in simulating the concentration of TDS even after modification was probably related to the local errors in process of simulating other fluxes as observed in^68^. The other possible sources of overestimation and underestimation of the model simulations were subjected to uncertainties of the input data and observed data. For example, because the data were collected once or twice in each month while the simulations were at daily time step, such a low frequent sampling might miss the peak or valley of nutrient concentrations.”

now reads:

“The TDS module for groundwater quality used the TDS concentrations from the selected groundwater monitoring stations in the ARB. The calibrated TDS parameters with specific ranges were presented in Table 1. Daily TDS concentrations were calibrated by adjusting the parameters related to groundwater. The model performance evaluation criteria reported by ^62^ were used for the daily nutrient simulation as a guideline in evaluating the model performance for the daily TDS concentrations. Figure 4 shows that for the whole period of simulation, R^2^, NSE and PBIAS values were found to be good to very good during calibration while they were satisfactory to good during the validation (Table 2). Generally, the overall model performance for the daily TDS concentration simulations in groundwater of the ARB shows that the model could capture the observed concentrations. When comparing the calculated and observed concentrations (Fig. 4), the simulated TDS concentrations were also acceptable as both show similar trends. Yet, local inconsistencies were noticed in the river basin. The highest percentage of overestimation and underestimation of TDS in the calibration dataset occurs for high concentration observations and probably reflects the SWAT model’s representation of high level concentrations^67^, which may cause errors in the process of calculating TDS in the SWAT model^68^. The other possible sources of overestimation and underestimation of the model simulations originate from uncertainties in the input data and observed data. For example, the frequency of data collection varied from once or twice per month to once every few years. In contrast, the simulations used a daily time step. The low frequency of sampling might miss the peak or valley of nutrient concentrations.”

“The TDS is indicator of the availability of total dissolved salt and other parameter that affect the groundwater quality. The availability of high electrical conductivity and observed TDS in the groundwater indicate a downward movement of leachate into the groundwater^69,70,71^. The TDS loads of the groundwater in the study area ranges 528.05 mg/L to 412.2 mg/L at ABG0215416 and ABG0220481 stations, respectively. The TDS of groundwater samples ranges 289–862 mg/L at ABG0215416 station, while the daily TDS ranges 321–533 mg/L at ABG0220481 station (Fig. 4). High concentrations of TDS in the groundwater reduce the palatability of water in the ground and may cause gastrointestinal pain in human stomach and emetic effects upon transits^72^. As TDS is an important indictor for evaluating the quality of groundwater, high level of TDS typically shows hard water and might require groundwater treatment to WHO standard with the limited concentrations of TDS is 500 mg/L^72^.”

now reads:

“Total Dissolved Solids is an indicator of the availability of total dissolved salt and other constituents that affect groundwater quality. High TDS concentrations in groundwater can occur naturally, and may also indicate a downward movement of leachate into groundwater^65,69,70,71^. The average TDS concentrations in groundwater in the study area are 143 mg/L and 1421 mg/L at IOR-KRL-03(ss) and IOR-KRL-03(ss) stations, respectively. The TDS of groundwater samples ranges from 120–182 mg/L at the IOR-KRL-03(ss) station, while the TDS ranges from 1380–1500 mg/L at the IOR-KRL-04(ss) station (Fig. 4). High concentrations of TDS in groundwater reduce the palpability of water for drinking and may cause gastrointestinal pain and emetic effects in humans^67^. TDS is an important indicator for evaluating the quality of groundwater. High levels of TDS typically occur for hard water and might require groundwater treatment to decrease concentrations below 500 mg/L^67^.”

The subheading ‘Groundwater quality analysis and nutrient fluxes’ has been renamed to ‘Groundwater quality analysis and nutrient concentrations’.

Under the subheading ‘Groundwater quality analysis and nutrient concentrations’,

“Analysis of observed against simulated in the ARB groundwater condition helps to identify sources of errors across the two monitoring stations of the river basin. Daily observed nitrate concentrations vary significantly across the area (Fig. 3). Concentrations of NO_3_^−^ in the groundwater have been highly affected by emissions of both point and non-point sources in different watersheds across the world. To assess pollution sources and quantify the loads of NO_3_^−^ entering the whole river basin, NO_3_^−^ load in the groundwater has been simulated at various hydrological response unit. The simulation periods were chosen based on the availability of point sources information. The long-term daily average concentrations of nitrate in the period 2003 attaining the maximum values in the ABG0215416 station, in which the mean value found to be 0.46 mg/L. While in station, the daily mean value reached the lowest values in the 0.012 mg/L for the year 1987. On the other hand, at station ABG0220481 the long-term daily average NO_3_^−^ concentrations recorded a maximum value of 0.29 mg/L during the year 2003 while attained the lowest value of 0.01 mg/L during the year 1989. Nitrate is the main component of total nitrogen (which accounts for around 70% of TN) and the concentrations of both nitrate and total nitrogen decrease with increasing the size of the river. Generally, the concentrations of nitrate were well captured in all the groundwater monitoring stations, although some overestimations were observed at some points at ABG0215416 monitoring stations (Fig. 3) and some underestimations were found at ABG0215416 and ABG0220481 monitoring stations.”

now reads:

“Analysis of observed against simulated nitrate and TDS concentrations in ARB groundwater helps to identify sources of errors across two groundwater monitoring stations within the river basin. Observed nitrate concentrations vary significantly across the area (Fig. 3). Concentrations of NO_3_^−^ in groundwater have been highly affected by emissions of both point and non-point sources in different watersheds across the world^71^. To assess pollution sources and quantify the loads of NO_3_^−^ entering the whole river basin, NO_3_^−^ load to groundwater has been simulated at various hydrogeological response units. The simulation periods were chosen based on the availability of point-source information. The long-term daily average concentrations of nitrate in groundwater attained maximum values of 0.59 mg/L at the IOR-KRL-03(ss) station during 2013. The long-term daily average concentrations of nitrate in groundwater reached a minimum of 0.025 mg/L in the year 2014. On the other hand, at station at IOR-KRL-04(ss) the long-term daily average NO_3_^−^ concentrations recorded a maximum value of 0.1 mg/L during the year 2015, and a minimum value of 0.013 mg/L in most observation years. Generally, the concentrations of nitrate were well captured in all the groundwater monitoring stations, although some overestimations were observed at some points at IOR-KRL-04(ss) monitoring station (Fig. 3) and some underestimations were found at IOR-KRL-03(ss) and IOR-KRL-04(ss) monitoring stations.”

“In fact, soil denitrification caused excessive NO_3_^−^ simulation could improved by varying the river basin parameters. However, the existing SWAT model could not represent perfectly the seasonal difference of nitrate. After carefully extending the model, the findings of this study highlight the essential to improve spatial representation of nitrification in the groundwater where its dependability is restricted by setting appropriate parameters at watershed level.”

now reads:

“The simulation of NO_3_^−^ removal by soil denitrification could be improved by varying the river basin parameters. However, the existing SWAT model could not represent perfectly the seasonal difference in nitrate concentrations. After carefully extending the model, the findings of this study highlight the need to improve spatial representation of nitrate concentrations in groundwater and in the parameters that influence nitrate concentrations at the watershed scale.”

In the Concluding remarks section, groundwater pollution was attributed to fertilizers, manure and industrial wastes and claims that impacts of point and non-point sources were detected, but these were not findings of the study. This section incorrectly implied that there is not a monitoring network in the river basin.

As a result,

“Groundwater is the precious natural resource for the existence of life on earth. However, various factors, such as soil properties, crop growth, industrial wastes, and agricultural management, influenced its quality. Due to development of industries, agriculture, and fisheries, increasing water uses put several burdens on groundwater quality influencing the ecosystem of the Athabasca River Basin. Particularly, the use of fertilizers, manure, and industrial wastes significantly contributed to the pollution of groundwater. It is necessary to perform optimal management of ground water resources in the basin. A state-of-the-art groundwater quality modelling at the ARB is recognized as a vital spot where further in-depth studies may be required to offer valuable insights related to groundwater condition and nutrient processes at the various spatio-temporal scale (i.e. site and region, and daily, monthly and annually). This can better support the nutrient monitoring network in the river basin management and harmonize monitoring concentrations of nutrient in the ARB. Therefore, SWAT model results could support the development of indicators of water quality parameters with groundwater in a nexus thinking approach. However, the accuracy of SWAT predictions is limited by data availability and structure of the model. This would result in some sort of errors during modeling process in the river basin. For example, the highest percentage of overestimation and underestimation performance for the TDS in the calibration dataset probably reflected the poor representation of SWAT model to high-level concentrations and uncertainties of the input data and observed data, which in turns may cause errors in estimating the TDS in the SWAT model. With the advent of monitoring network, quality observed input data would be available for better calibration and validation of the model. Furthermore, the quality data would improve the representation of the processes and pathways, which account for groundwater pollution and therefore evaluate the effect of management practices to support the implementation of the best management practices.

In this study, we extended the existed SWAT model to better represent water carrying capacity of quality parameters in modelling groundwater quality (i.e. NO_3_^−^ and TDS). Groundwater quality modelling could detect the impact of point and nonpoint sources of pollutants on groundwater. Elevated sources of NO_3_^−^, and TDS could be percolated into the groundwater in the form of lateral flow. A systematic calibration and validation of the SWAT model has been performed to compare the observed groundwater NO_3_^−^ and TDS fluxes in the ARB. The results reviled that the new groundwater quality model in the SWAT is able to capture the daily nutrient concentrations of groundwater. The simulated results agree with the observed data with satisfied and good performance for both stations with an averaged *R*^2^ of 0.77 for nitrate during calibration and 0.68 during validation. Thus, the process-based hydrologic groundwater quality model is an effective tool in simulating the groundwater quality dynamics (NO_3_^−^ and TDS) for sustainable groundwater and surface water management in the river basin.”

now reads:

**Figure 1:**
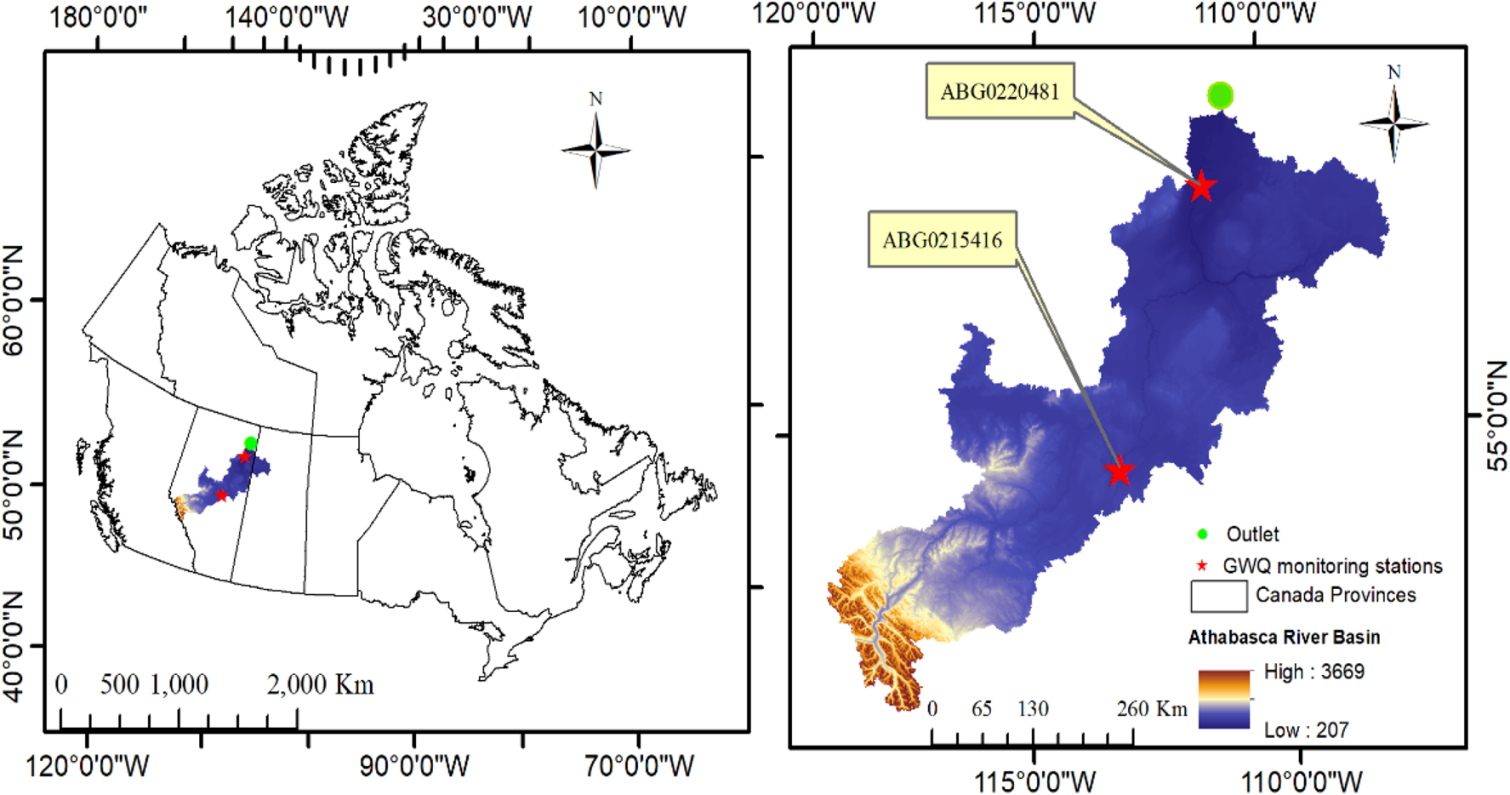
Geographical location of Athabasca River Basin (ARB), Canada. The DEM of the ARB shows the two water quality monitoring stations used for model calibration for this study. The map was generated using GIS & RS (https://www.arcgis.com/index.html).

“Groundwater is a precious natural resource supporting the existence of life on earth. However, various factors, such as soil properties, crop growth, industrial wastes, and agricultural management, can influence its quality. Development of industries and agriculture increase water use, which puts pressure on groundwater quality and may, in turn, influence the ecosystem of the Athabasca River Basin. Particularly, the use of fertilizers, manure, and industrial wastes may contribute to the pollution of groundwater and connected surface water environments. It is necessary to perform optimal management of groundwater resources in the basin.

State-of-the-art groundwater quality modelling at the ARB is recognized as a vital component of groundwater management, where further in-depth studies may be required to offer valuable insights related to groundwater condition and nutrient processes at the various spatio-temporal scales (i.e. site and region, and daily, monthly and annually). This can better support nutrient monitoring networks for river basin management, and enhance understanding of changing nutrient concentrations in the ARB. The SWAT model results could support the development of indicators for groundwater quality parameters, and also support integrated surface water and groundwater management. However, the accuracy of SWAT predictions is limited by data availability and structure of the model. This may result in errors while modeling processes in the river basin. For example, the highest percentage of overestimation and underestimation for TDS in the calibration dataset probably reflected the poor representation of SWAT model to high concentrations, as well as uncertainties of the input data and observed data, which in turn may cause errors in estimating groundwater TDS in the SWAT model. With continued monitoring of the groundwater network at regular frequencies, high quality input data would be available for better calibration and validation of the model. Furthermore, additional data would improve the representation of processes and pathways controlling groundwater pollution and therefore allow evaluation of the effect of land and water management practices to support the implementation of the best management practices.

In this study, we extended the existing SWAT model to improve modelling of groundwater quality (i.e. NO_3_^−^ and TDS). A systematic calibration and validation of the SWAT model has been performed to compare the observed groundwater NO_3_^−^ and TDS concentrations in the ARB. The results revealed that the new groundwater quality model in the SWAT is able to capture the daily nutrient concentrations in groundwater. The simulated results agree with the observed data with satisfactory and good performance for both groundwater monitoring stations as per the model performance measures. Thus, the process-based hydrologic groundwater quality model is an effective tool in simulating the groundwater quality dynamics (NO_3_^−^ and TDS) for sustainable groundwater and surface water management in the river basin.”

Furthermore, Figures 1, 3, 4 and 5 and the accompanying legend were incorrect where the Figures contained the data for monitoring stations IOR-KRL-03 (ss) and IOR-KRL-04 (ss). These stations were replaced with the stations ABG021546 and ABG022048. The original Figure 1, 3, 4 and 5 and accompanying legend appear below.

**Figure 3:**
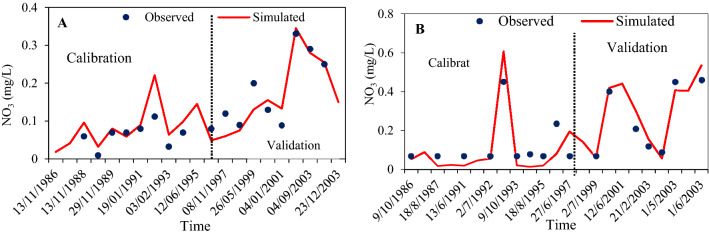
Comparison of observed and simulated groundwater quality parameter (NO_3_/mg/L) at ABG0215416 (**A**) and ABG0220481 (**B**) monitoring stations.

**Figure 4:**
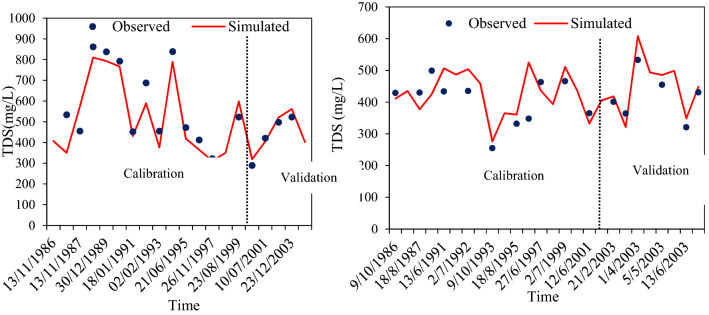
Comparison of observed and simulated groundwater quality parameter (TDS/mg/L) at ABG0215416 (**A**) and ABG0220481 (**B**) monitoring stations.

**Figure 5:**
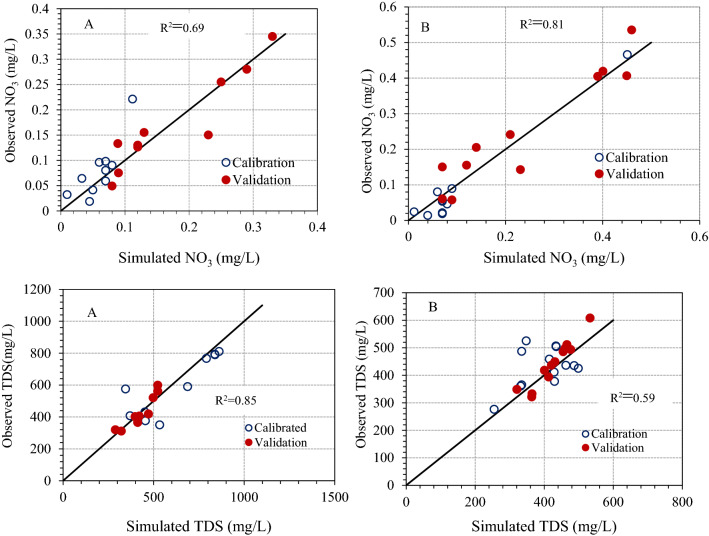
Scatter plot comparison between of daily simulated and observed groundwater quality parameters at ABG0215416 (**A**) and ABG0220481 (**B**) monitoring stations.

Lastly, the work of Arnold et al. (2012) was omitted from the Reference List. References 40 and 41 were incorrectly given as:

40. AWC. State of the watershed report: Phase 3—Water quantity and basic water (2013).

41. Quality in the Athabasca Watershed. Athabasca Watershed Council.

The correct references are listed below:

40. AWC. State of the Watershed Report: Phase 3–Water Quantity and Basic Water Quality in the Athabasca Watershed. Athabasca Watershed Council. (2013).

41. Arnold, J.G., *et al.* SWAT: model use, calibration, and validation. *Am. Soc. Agric. Biol. Eng*. **55** (4), 1491–1508 (2012).

These Reference are now cited at the relevant points in-text.

The original Article has been corrected.

